# The effect of providing feedback on inhaler technique and adherence from an electronic audio recording device, INCA®, in a community pharmacy setting: study protocol for a randomised controlled trial

**DOI:** 10.1186/s13063-016-1362-9

**Published:** 2016-05-04

**Authors:** Susan Mary O’Dwyer, Elaine MacHale, Imran Sulaiman, Martin Holmes, Cian Hughes, Shona D’Arcy, Viliam Rapcan, Terence Taylor, Fiona Boland, Sinthia Bosnic-Anticevich, Richard B. Reilly, Sheila A. Ryder, Richard W. Costello

**Affiliations:** School of Pharmacy and Pharmaceutical Sciences, Trinity College Dublin, Dublin 2, Ireland; Pharmacy Office, Boots Retail Ireland Limited, Nangor Road, Dublin 12, Ireland; Clinical Research Centre, Smurfit Building Beaumont Hospital, RCSI, Dublin, Ireland; Department of Bioengineering, Trinity College Dublin, Dublin 2, Ireland; Royal College of Surgeons in Ireland, 123 St Stephen’s Green, Dublin 2, Ireland; Woolcock Institute of Medical Research, University of Sydney, Sydney, NSW Australia; Department of Respiratory Medicine, Royal College of Surgeons in Ireland, 123 St Stephens Green, Dublin 2, Ireland

**Keywords:** Adherence, INCA® electronic monitor, Cluster randomised trial, Inhaler technique, Community pharmacy, Patient education

## Abstract

**Background:**

Poor adherence to inhaled medication may lead to inadequate symptom control in patients with respiratory disease. In practice it can be difficult to identify poor adherence. We designed an acoustic recording device, the INCA® (INhaler Compliance Assessment) device, which, when attached to an inhaler, identifies and records the time and technique of inhaler use, thereby providing objective longitudinal data on an individual’s adherence to inhaled medication. This study will test the hypothesis that providing objective, personalised, visual feedback on adherence to patients in combination with a tailored educational intervention in a community pharmacy setting, improves adherence more effectively than education alone.

**Methods/design:**

The study is a prospective, cluster randomised, parallel-group, multi-site study conducted over 6 months. The study is designed to compare current best practice in care (i.e. routine inhaler technique training) with the use of the INCA® device for respiratory patients in a community pharmacy setting. Pharmacies are the unit of randomisation and on enrolment to the study they will be allocated by the lead researcher to one of the three study groups (intervention, comparator or control groups) using a computer-generated list of random numbers. Given the nature of the intervention neither pharmacists nor participants can be blinded. The intervention group will receive feedback from the acoustic recording device on inhaler technique and adherence three times over a 6-month period along with inhaler technique training at each of these times. The comparator group will also receive training in inhaler use three times over the 6-month study period but no feedback on their habitual performance. The control group will receive usual care (i.e. the safe supply of medicines and advice on their use). The primary outcome is the rate of participant adherence to their inhaled medication, defined as the proportion of correctly taken doses of medication at the correct time relative to the prescribed interval. Secondary outcomes include exacerbation rates and quality of life measures. Differences in the timing and technique of inhaler use as altered by the interventions will also be assessed. Data will be analysed on an intention-to-treat and a per-protocol basis. Sample size has been calculated with reference to comparisons to be made between the intervention and comparator clusters and indicates 75 participants per cluster. With an estimated 10 % loss to follow-up we will be able to show a 20 % difference between the population means of the intervention and comparator groups with a power of 0.8. The Type I error probability associated with the test of the null hypothesis is 0.05.

**Discussion:**

This clinical trial will establish whether providing personalised feedback to individuals on their inhaler use improves adherence. It may also be possible to enhance the role of pharmacists in clinical care by identifying patients in whom alteration of either therapy or inhaler device is appropriate.

**Registration:**

ClinicalTrials.gov NCT02203266.

**Electronic supplementary material:**

The online version of this article (doi:10.1186/s13063-016-1362-9) contains supplementary material, which is available to authorized users.

## Background

Asthma and chronic obstructive pulmonary disease (COPD) are common, chronic respiratory diseases [1, 2]. Both conditions are characterised by symptoms such as wheeze, breathlessness and airflow limitation [3, 4] which vary in intensity from patient to patient.

Appropriate pharmacological treatment, used correctly, can help to reduce symptoms and improve quality of life [3, 4]. Inhaled medications are commonly prescribed but can be difficult to use and this can lead to technique errors [5, 6] that may have both direct costs such as medication waste, and indirect costs such as increased use of other health care resources as a consequence of poor symptom control [7].

Community pharmacist-delivered interventions have been shown to improve inhaler technique [8, 9], inhaled mediation adherence rates [10] and therapeutic outcomes [11–14] for adult patients with asthma [8, 9, 11–14] and COPD [8, 10]. However, adherence rates in reported studies are frequently either based on patient self-report which has been shown to be unreliable [15, 16] or calculated from pharmacy databases containing prescription refill data which provide information on medication obtained but not on how such medication is used in practice [17, 18].

We developed a device, INCA®(INhaler Compliance Assessment), which makes a digital acoustic recording of an inhaler being used [19]. In this study the device is attached to a salmeterol/fluticasone Diskus® (Seretide Diskus®, also known as Seretide Accuhaler®) inhaler. When a patient opens their Diskus® inhaler (by sliding a thumbgrip to expose the mouthpiece) the INCA® device switches on and begins to audio record. The recordings are analysed using automated signal processing techniques, thus providing an objective assessment of both time and technique of inhaler use [19].

Participants’ adherence to their inhaled medication will be described in terms of both attempted and actual adherence rates. Where the participant opens their Diskus® inhaler, initiating an audio recording, this is classed as an attempt to take the inhaled medication. The pattern of attempts over a defined period will provide an ‘attempted’ rate of adherence. Analysis of the associated acoustic recordings allows for detection of correct inhaler technique, which we take to indicate that the participant actually received the dose they attempted to take. Thus, the pattern of attempted doses with correct technique yields the ‘actual’ rate of adherence.

We hypothesise that the INCA® device, when used as part of a community pharmacist-delivered adherence-focussed educational consultation, can support medication management in patients with respiratory disease.

## Methods

### Study setting

This is a prospective, cluster randomised, parallel-group, multi-site study comparing two pharmacist-delivered strategies to optimise inhaler technique and adherence in respiratory patients in the community setting. The study will be conducted in a chain of 77 community pharmacies operating in the Republic of Ireland. Pharmacies are eligible for inclusion in the study if they have patients who are prescribed a salmeterol/fluticasone Diskus® inhaler and if those patients have regularly attended the pharmacy to collect a prescription in the 6 months prior to enrolment. The study period is from 2014 with ongoing recruitment.

### Participants

Adult patients of both sexes aged 18 years or older who use, or are capable of using, a salmeterol/fluticasone Diskus® inhaler and are in possession of a current valid prescription for the same, are eligible to enrol on the study.

### Inclusion and exclusion criteria

In order to meet inclusion criteria for the study patients must be capable of understanding, and willing to provide, voluntary informed consent before any protocol-specific procedures are performed; be capable of understanding and complying with the requirements of the study protocol; demonstrate a willingness to attend for all required visits; be able and willing to use inhaled medication and have a history of regular attendance at the pharmacy in which they are recruited, receiving at least three prescriptions for any medication filled in the prior 6 months .

Patients are excluded from the study if they have a known sensitivity to salmeterol/fluticasone, if their physician has indicated that they will not be continuing to receive salmeterol/fluticasone Diskus® over the 6-month study period or if they have used any investigational product or device within the 3 months prior to enrolment.

### Study design

**Fig. 1:**
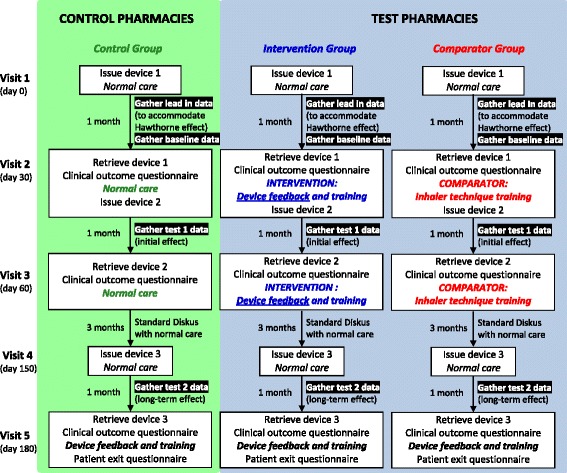
INCA® Pharmacy Study – overview of study flow and design

A cluster randomised study has been chosen in order to minimise the risk of contamination of knowledge by the pharmacists across the three study groups. The study flow is indicated in Fig. 1 (see Additional file 1).

Patients identified in the community pharmacy, who meet the inclusion and do not violate the exclusion criteria, will be invited to participate in the study and are provided with information about the trial in the form of a participant information leaflet. On enrolment, consent will be obtained and recorded. All study visits are conducted by a registered pharmacist.

At the initial visit each participant’s age, sex, dose of salmeterol/fluticasone, duration of taking this dose and details of concomitant medications will be recorded on paper-based case report forms which have been developed specifically for this study and piloted in a sample of three pharmacies. The pharmacist will record the Peak Expiratory Flow Rate (PEFR) as measured by an electronic peak flow meter (eMini-Wright®, Clement Clarke International, Harlow, England). The St. George’s Respiratory Questionnaire (SGRQ) [20], a validated tool used to assess quality of life in patients with diseases causing airway obstruction, will be completed by the participant. A history of inhaled therapy (salmeterol/fluticasone and short-acting beta agonists), steroid and antibiotic usage over the prior 6 months will be obtained from the participant’s patient medication record in the pharmacy. Participants will be asked to identify an aspect of their life affected by their respiratory condition that they would like to improve. This ‘breathing-related goal’ will be recorded and improvement will be monitored through the study period. The purpose of this is to provide an additional focus for the participant via which a tangible and personalised measure of any symptom improvement can be noted. Patient reported clinical diagnosis (asthma or COPD), smoking status and health pay status will also be recorded for all participants.

**Fig. 2:**
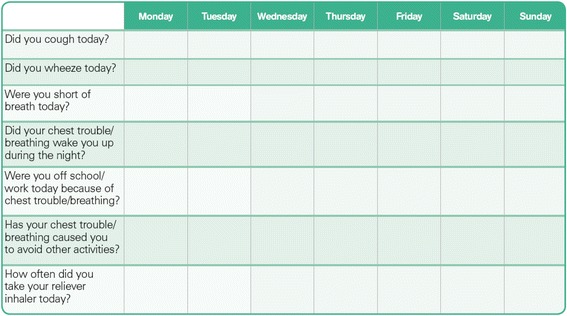
Extract of participant respiratory diary

The participants will receive a salmeterol/fluticasone Diskus® inhaler with an INCA® device attached for use twice per day as prescribed. This inhaler will be provided free of charge to all participants, regardless of their health pay status, with the relevant professional service being paid by the patient in the usual manner. For all participants, the INCA® device is used to capture data on adherence and inhaler technique. Participants in the intervention and comparator groups will be asked to record their peak expiratory flow with the eMini-Wright® electronic monitor twice daily. In the intervention and comparator groups participants are also provided with a paper-based diary for recording daily symptoms, medication usage and health care utilisation. This diary, based on the Asthma Society of Ireland’s peak flow diary [21], has been developed specifically for this study and piloted in a sample of seven patients. An extract of the diary is provided in Fig. 2 (see Additional file 1).

After the initial visit (visit 1), follow-up visits are scheduled 30 (visit 2), 60 (visit 3), 150 (visit 4) and 180 (visit 5) days later. At visits 2, 3 and 5 the participants return their inhalers with the INCA® devices. Visit 4 is a dispensing visit. At each of these visits, for control group participants, changes in medications (including new medications) are recorded as is progress towards the participant’s ‘breathing-related goal’. For intervention and comparator group participants this information is also noted along with the SGRQ responses. Assessment of inhaler technique, using an inhaler technique checklist and inhaler technique training based on assessment observations, is given to both intervention and comparator participants.

### Interventions

#### Intervention group: feedback using recordings from the INCA® device

Participants in the intervention group will receive the INCA® intervention. These participants will receive feedback on their own inhaler use, with personalised information on their technique and timing of use of the salmeterol/fluticasone Diskus® inhaler as recorded on the INCA® device at day 30 (visit 2), day 60 (visit 3) and day 180 (visit 5). Utilising a structured review, the pharmacist will aim to identify barriers to good adherence and support patients to improve habit of use where necessary. Remediation of errors of inhaler use identified through assessment of participants’ inhaler technique using a Diskus® inhaler technique checklist, the ‘Inhaler Proficiency Schedule’ (IPS) [22], will also be conducted.

### Comparator group: inhaler technique education

The comparator for this study is current best practice in the community pharmacy setting, defined for this study as an assessment of the participant’s inhaler technique using a Diskus® inhaler technique checklist (IPS) [22] and a physical demonstration of optimal technique by the pharmacist followed by demonstration of the same by the participant until device mastery is achieved.

### Control group: usual care

In order to control for trial participation effects a control group is included the study design. Participants in the control group will receive no intervention other than usual care (i.e. the safe supply of medicines and advice on their use). Review of inhaler technique, as initiated by the pharmacist where deemed necessary, or as requested by the participant, is a feature of usual care but its provision is not a standard or structured intervention in the control group.

Upon exiting from the study at visit 5, all participants will receive personalised feedback on their Diskus® use based on the INCA® device recordings.

### Objectives

The objective of this study is to assess whether providing personalised feedback on adherence and technique errors will lead to better adherence and clinical outcomes for participants than current best practice.

### Primary outcome

The primary study objective is to determine whether there is at least a 20 % greater rate of actual adherence in the INCA® feedback group (‘intervention group’) compared to the current best practice group (‘comparator group’) post intervention. Thus, the primary outcome measure will be the rate of participant adherence to their inhaled medication, defined as the proportion of correctly taken drugs at the correct time relative to the prescribed interval (defined for this study as the dosing interval ±25 %). To accommodate for potential Hawthorne effect comparisons of the rate of actual adherence at 2 months and at 6 months will be performed.

### Secondary outcome analysis will include

Comparison of the proportion of patients in each group who achieved good actual adherence (defined for this study as ≥80 %) as well as the number progressing to good technique (≥80 % of all doses attempted by the patient classified as correct technique) at both 2 and 6 months will be assessed. Changes in patterns of adherence in the intervention and comparator groups will also be reviewed by looking at the morning and evening habit of inhaler use, error rates, overdose rates, intervals and attempted rates of adherence.

The PEFRs, SGRQ responses and rescue medication (inhaler, steroid, antibiotic) use of the intervention and comparator groups will be compared, as will levels of reported symptoms (as noted in the participants’ respiratory diaries) and improvement in participants’ ‘breathing-related goals’.

Prescription medication dispensing records will be reviewed to provide a further assessment of adherence (as defined by the medication possession rates and/or the proportion of days covered method) pre, during and post the study, and adherence measured in this way will be compared across the intervention and comparator groups; it will also be compared with actual adherence rates determined by the INCA® device.

### Sample size calculation

Studies in the pharmacy setting have identified rates of baseline rates of attempted adherence amongst asthma patients of below 0.6 [12]. In the primary care setting poor inhaler technique amongst patients with respiratory disease has been identified in preliminary studies conducted using the INCA® device (47 %, standard deviation 33.0). A pre-intervention actual adherence rate of 0.5 at the end of the first month is, therefore, assumed. The primary endpoint is the rate of actual adherence at the end of the study period. A comparison between adherence rates in the intervention and comparator groups will be conducted at the end of months 1, 2 and 6. The sample size is thus dictated by comparisons to be made between these two groups.

It is hypothesised that the intervention group will get closer to the commonly reported level of ‘good’ adherence (≥0.8) [23] improving by 0.2. Adherence in the comparator group may improve as a result of the educational intervention received and an assumed improvement of 0.05 in this group over the study period has been incorporated into the sample size calculation. An intra-class correlation coefficient of 0.025 and a loss to follow-up of 10 % (as observed in a recent similar study conducted in the pharmacy setting [14]) are assumed. With a power of 0.85 at the 0.05 significance level to detect a 0.2 difference in actual adherence between the two groups, a sample size of 75 participants across 25 clusters in each of the intervention and comparator groups is required.

### Recruitment

Patients attending the study pharmacy who are prescribed a salmeterol/fluticasone Diskus® inhaler will be identified via prescription medication records. On attendance to collect their medication in the pharmacy patients will be invited to participate in the study. Interested patients will be screened for eligibility by the pharmacist and if eligible will be provided with information about the study and a copy of the patient information leaflet. Voluntary informed consent will be obtained for all recruited participants.

### Randomisation and allocation

Pharmacies are the unit of randomisation and on enrolment to the study they will be allocated by the lead researcher to one of the three study groups (intervention, comparator or control groups) using a computer-generated (Microsoft Excel® 2013) list of random numbers. In order to control for trial participation effects 10 pharmacies will be randomly allocated to the control group. Remaining pharmacies will then be randomly allocated to either the intervention or comparator group in a 1:1 ratio. Given the nature of the intervention neither pharmacists nor participants can be blinded. All participants are aware that data on adherence are being collected for analysis and a lead-in period is incorporated into the study design to account for a potential Hawthorne effect.

### Pharmacist training

Pharmacists will deliver the intervention allocated to the pharmacy in which they practise and they will receive training specific to that intervention. Training will consist of one 1.5-hour face-to-face workshop with a respiratory physician, nurse specialist and pharmacist educator/researcher where they will be provided with an overview of the study and trained to provide education on inhaler technique and medication adherence. A distance learning study guide specific to each study arm and outlining all study-related procedures will also be completed. Throughout the study pharmacists will be supported by a lead pharmacy researcher who will be available via phone or email to answer any queries they may have.

### Data collection, management and analysis

#### Data collection

Pharmacists completing data collection are trained to understand the importance of robust data collection, are provided with a study guidance pack outlining all relevant data collection procedures and sign a declaration indicating their agreement to keep complete and accurate records. The accuracy, completeness and progress of data will be overseen by a lead researcher in the pharmacy chain where the study is being conducted. This researcher, who will have no role in participant recruitment or in conducting any study-related procedures, will conduct visits to the study pharmacies to check compliance with the protocol.

Every reasonable effort to follow up all enrolled participants for the entire 6-month study period will be employed. At each study visit an appointment for the next visit will be scheduled and non-attenders will be contacted to re-schedule as required. The lead pharmacist researcher will monitor recruitment and retention and target counselling and training interventions to pharmacy sites where retention is a challenge. At any point in time, a study participant is free to withdraw their consent from the study. A patient can be withdrawn from the study by the investigator if necessary, based on clinical assessment of adverse events, or in the event of early discontinuation of the study. In the case of withdrawing from the study, consultation will be completed where possible and their adapted inhaler, peak flow meter and diary will be collected.

### Safety reporting

Safety testing and a comprehensive risk assessment have been conducted in an effort to minimise potential hazards associated with the clinical investigation of this device. The INCA® device will be securely fixed to the outer casing of the inhaler. It does not interfere in any way with the inhaler’s mechanism of drug delivery. However, in the event of any safety issues arising adverse events and serious adverse events will be recorded in the case report form and evaluated by the lead pharmacy investigator. The principle investigator, ethics committees and the Health Products Regulatory Authority (HPRA) will be notified of any serious adverse events that occur. In addition, information about device-related adverse events will be collected and reported to the manufacturer of the device and the HPRA. Drug-related serious adverse events will be reported to the manufacturer of the drug.

### Data management

Paper-based case report forms will be pseudonymised with a unique patient identifier code. On study completion or withdrawal, data from these report forms will be input by the lead researcher into an electronic, password-protected database. Participant files will remain locked in a secure and accessible place, in a manner consistent with local data protection requirements, and maintained in storage for a period of 3 years post termination of the study.

Audio data will be uploaded to a secure server, access to which is by individual user name and password. Individual pharmacists will not have access to this database. The tool has an inbuilt audit trail that records, and can display, details of additions or changes made to data either on a by-user or a by-patient basis.

### Analysis of the audio data

The INCA® device is a Conformité Européene (CE)-marked device which is manufactured by Vitalograph, Ennis, Ireland. Digital recordings from the INCA® device are analysed as previously described [19]. The files are uploaded to a secure server and analysed using signal processing methods. The sensitivity and specificity details of the signal processing algorithm have been published [24]. The algorithm identifies each audio file as one representing either correct or incorrect inhaler use as well as automatically classifying any technique errors identified. Two independent human raters also over-read all files from all participants in order to validate the data. Comparison is made between the automated and human classification as well as between the classifications of the two independent raters. In the event of any disagreement, review by a third over-reader yields the final classification by majority decision. The final classification is then used in the calculation of the actual adherence. The human raters are independent of any patient care during the trial and are blinded to the randomisation status of the patient. Critical inhaler errors which can occur include failure to prime the inhaler with drug, exhalation into or near the mouthpiece after priming but before inhalation, failure to achieve an adequate flow rate and the presence of multiple inhalations indicating inadequate breath-holds. Non-critical errors such as not holding the device level are not recorded.

### Statistical methods

Statistical analysis will be conducted using IBM SPSS Statistics for Windows, Version 21.0. (Armonk, NY, USA: IBM Corp). Descriptive statistics will be used to evaluate differences in demographic characteristics, clinical condition, medication profile, exacerbation history and quality of life scores between study groups. Categorical variables will be expressed as frequencies or percentages and quantitative variables as means and standard deviations or medians and interquartile ranges.

The primary analysis will be carried out using multilevel modelling (such as mixed linear effects modelling) with the individual as the unit of analysis and controlling for the effects of clustering and baseline differences. An intention-to-treat analysis will be conducted. A secondary, per-protocol analysis will also be conducted. This form of analysis includes only those participants who completed the treatment protocol originally allocated, providing results on the efficacy of the trial.

The proportion of patients achieving good actual adherence (≥80 %) and the proportion progressing to good technique (≥80 % of all doses attempted by the patient classified as correct technique) will be analysed using a random-effects logistic regression with the individual as the unit of analysis and the pharmacy included as the random effect, to control for the effects of clustering.

Changes in patterns of adherence will also be investigated. PEFR, rescue medication use and reported symptoms will be described and used as an indicator of the number of exacerbations per patient which will, in turn, be compared between the groups. Additionally, differences in the SGRQ responses will be examined. For all analyses stratification of patients by diagnosis will be attempted but consideration will be given to the ability to make inferences based on patient numbers.

A full statistical analysis plan will be written by an independent statistical team prior to any analysis being undertaken. Data will be reported in line with the Consolidated Standards of Reporting Trials (CONSORT) 2010 Statement [25] as well as its extension to cluster randomised trials [26] and non-pharmacological treatment [27].

## Discussion

The aim of this study is to evaluate the extent to which a community pharmacist-delivered intervention combining monitored adherence with repeated personalised education can improve inhaler technique and adherence in patients with respiratory disease. This is the first community pharmacy-based study to use a technology that objectively assesses appropriate use of inhalers both in terms of technique of use as well as time of use, permitting longitudinal evaluation of patients’ habitual performance when not under direct visual observation. This technology incorporates a novel electronic device, automated algorithms and feedback tools including graphical representations of adherence, facilitating personalised feedback provided by the pharmacist.

Pharmacists are increasingly involved in comprehensive medication management [28]. In order to optimise clinical outcomes for their shared patients close collaboration between pharmacists and physicians is likely to be required [29]. Provision of high-quality clinical recommendations that improve patient outcomes is important in establishing trust [30], a key component of successful pharmacist/physician collaboration [29, 31]. Collecting objective longitudinal information on inhaler adherence may support the formulation by the pharmacist of such recommendations, thus supporting the collaborative clinical decision-making process. It is expected that some participants may become fully adherent, with others continuing to demonstrate variable or poor adherence over the study period. Combining this information with clinical measures such as PEFR data and exacerbation rates may support inter-professional decision-making for individual patients by potentially distinguishing between patients who would benefit from further interventions to promote adherence as opposed to those in whom therapy adjustment or change of inhaler device may be more appropriate.

As INCA records comprise a much richer dataset than other adherence measures that are routinely available for patients in primary care (primarily prescription refill and dose counter information), analysis of the nature, prevalence and persistence of timing and technique errors identified by the INCA® device should also lead to a deeper understanding of adherence and the barriers to effective Diskus® use, potentially facilitating development of proactive countermeasures for all patients. Analysis of the study data will also provide a means of assessing the reliability of conventional adherence measures in identifying and assessing adherence.

The study has a number of limitations. Patients with asthma and/or COPD are eligible to join the study meaning that there is a lack of sample homogeneity with respect to clinical condition. Stratification by condition at point of analysis will be attempted, but such analysis may lack sufficient power to make valuable inferences meaning that the study may be limited in its generalisability.

Patients requiring regular medication for the treatment of a chronic condition, such as asthma or COPD, are not required to register with an individual pharmacy in the Republic of Ireland meaning that they are free to move between pharmacies when collecting their monthly medication. To avoid the situation where patients collecting a prescription for a salmeterol/fluticasone Diskus® inhaler in a study pharmacy as a one-off occurrence are recruited to the study (leading to greater potential for attrition) the inclusion criteria specify that the patient must have had at least three prescriptions for any medication filled in the study pharmacy in the prior 6 months. Whilst the three prescriptions do not need to have been for a salmeterol/fluticasone Diskus® inhaler this nevertheless raises the possibility that patients with adherence rates of at least 0.5 at baseline will be recruited. However, given that adherence rates based on prescription records frequently overestimate adherence [32], and as the INCA device is designed to assess actual adherence (defined as the number of doses taken correctly at the correct time), we feel that this is justified.

As with other cluster randomised trials where allocation concealment is not possible there is a risk of selection bias [33, 34] both from the pharmacist and participant perspective. To minimise such bias pharmacists in each individual site are trained to recognise the importance of unbiased recruitment and recruitment to each site is monitored by the lead pharmacist researcher so that potential bias can be identified and addressed through further counselling and/or training as required.

There is a potential for loss to follow-up. To minimise these losses, participants not attending for study visits at scheduled times will be contacted by telephone a maximum of three times. Potential loss to follow-up has been accounted for in the sample size estimation and will be taken account of in the intention-to-treat analysis of study data.

### Trial status

Ongoing, currently recruiting participants.

### Ethical approval and consent to participate

The use of the INCA device in clinical investigation trial has been approved by the Irish Medicines Board (now Health Products Regulatory Authority) and the device is CE marked (CE0086). The study has been approved by Royal College of Surgeons in Ireland Ethics Committee (REC712b) and is registered as NCT02203266 on ClinicalTrials.gov (27 July 2014). The study will be conducted according to the principles of the Declaration of Helsinki (64th WMA General Assembly, Fortaleza, Brazil, October 2013), international standards of Good Clinical Practice, local regulatory requirements and this study protocol. In the event of any changes to the protocol a written application will be submitted to the Research Ethics Committee prior to implementation. Informed consent will be obtained from all participants.

### Publication of findings

It is intended to publish the results from this trial in scientific, medical and nursing journals and through other appropriate channels on completion of the trial. No confidential information about participants will be revealed in publications or dissemination of findings.

### Availability of supporting data

Not applicable.

### Consent to publish

Not applicable.

## Additional file

Additional file 1: SPIRIT 2013 Checklist. (DOC 121 kb)

## Abbreviations

HPRA: Health Products Regulatory Authority
INCA®: inhaler compliance aid
IPS: Inhaler Proficiency Schedule
PEFR: peak expiratory flow rate
SGRQ: St George’s Respiratory Questionnaire, respiratory quality of life score
